# Effects of Using a Smart Bassinet on the Mental Health of Military-Affiliated Pregnant Women: Protocol for a Randomized Controlled Sleep Health and Mood in Newly Expectant Military Mothers (SHINE) Trial

**DOI:** 10.2196/66439

**Published:** 2025-04-10

**Authors:** Michele L Okun, Jennifer L Payne, Lauren M Osborne, Leilani Feliciano, Andrew Lac

**Affiliations:** 1 University of Colorado Colorado Springs Colorado Springs, CO United States; 2 University of Virginia Charlottesville, VA United States; 3 Weill Cornell Medicine New York, NY United States

**Keywords:** maternal health, postpartum, pregnancy, sleep, infant, depression, anxiety, smart bassinet, intervention, prevention, military

## Abstract

**Background:**

Postpartum mood and anxiety disorders (PMADs) are higher among pregnant military service women (26%) and military spouses (12.2%) compared to the civilian population (10%-15%). This is partly due to military-specific factors, including deployment, which are known to increase risk. Important risk factors for PMADs include sleep disturbances, defined as sleep deprivation, insomnia, or poor sleep quality, which are more are common among military-affiliated pregnant women.

**Objective:**

This study describes a protocol for a new randomized controlled trial that aims to ameliorate the risk for PMADs through improving infant sleep or maternal sleep during the first 6 postdelivery months in a sample of military-affiliated women.

**Methods:**

This study is a 6-month, parallel-arm, randomized controlled trial. Pregnant women (N=342) in the third trimester will be randomized at 1:1 ratio to use a smart bassinet (SB) or a standard commercially available bassinet (HALO BassiNest Swivel Sleeper 3.0; traditional bassinet [TB]) for up to 6 months after delivery. Participants will have their infants sleep in the bassinet, complete monthly web-based questionnaires, and record sleep data with diary and actigraphy for both the participants and their infants for 1 week each postpartum month. Blood samples will also be collected at baseline (late pregnancy) and at 3 months and 6 months post partum to assess immune functioning. The primary outcomes for this study will be postpartum mood (depressive and anxiety symptoms) and infant and maternal sleep. In addition, we are evaluating whether SB has a significant impact on immune functioning—a marker that physiologically connects sleep and mood symptoms.

**Results:**

Recruitment for this study began in January 2025. Six separate mixed 2 (treatment vs control) × 6 (assessment period) multivariate analysis of variance and analysis of variance models will be conducted to test the hypotheses that SB will have a greater impact on infant and maternal sleep than TB, SB will be associated with a greater reduction in postpartum mood symptoms than TB, and immune system function will be less dysregulated in birthing individuals using SB compared to those using TB. Lastly, we will evaluate whether the elevated risk demonstrated by previously identified postpartum depression epigenetic biomarkers in the *TTC9B* and *HP1BP3* genes can be modified with an SB. We hypothesize that the elevated risk will be reduced in SB compared to that in TB.

**Conclusions:**

At the conclusion of this project, we will have gained a thorough understanding of the capability of SB to positively affect infant and maternal sleep compared to the traditional sleep arrangement and its impact on maternal mood through 6 months post partum in military-affiliated women. The promotion of sleep health in both mothers and infants may be an accessible and amenable method to prevent PMADs.

**Trial Registration:**

ClinicalTrials.gov NCT06544941; https://clinicaltrials.gov/study/NCT06544941

**International Registered Report Identifier (IRRID):**

PRR1-10.2196/66439

## Introduction

Postpartum mood and anxiety disorders (PMADs) are the most common and disabling complications of childbearing. They are often underdiagnosed and undertreated [1]. PMADs are recognized to seriously affect both mother and baby [2,3]. In fact, mental health conditions are a leading contributor to maternal morbidity, with suicide as a major cause of postpartum death. According to the Department of Defense, the number of females in the US military constituted 17.3% of the total force in 2021 [4]. Research indicates that the rate of PMADs in active-duty women can range from 20% to 26% [5-7], with slightly lower rates among military spouses at around 12.2%. Although these rates are similar to that reported in the civilian population (~10%-15%) [8-10], when other factors such as deployment are considered, the rates vary dramatically, reaching as high as 50% [11-16]. Women with spouses who are deployed have more frequent diagnoses of depressive disorders, sleep disorders, anxiety, acute stress reactions, and adjustment disorders compared to women without a deployed spouse. Despite these elevated risks, this segment of our population has been insufficiently evaluated as it pertains to PMAD risk and warrants further attention.

It is well appreciated that sleep disturbance is associated with both new and recurrent depressive and anxiety episodes in all populations, especially perinatal women [17-22]. Sleep disturbance often precedes the development of mood disorders [23,24]. Indeed, sleep issues lasting for at least 2 weeks increase the risk for the future development of mood disorders [25,26] and are a criterion for the diagnosis of depression in the Diagnostic and Statistical Manual of Mental Health Disorders, Fifth Edition, Text Revision (eg, major depressive disorder) [27]. Sleep disturbances, both in prevalence and severity, in military-affiliated women extend beyond what civilian women experience. Active-duty service members and their spouses experience unique stressors that often exacerbate sleep issues [28,29]. Despite this, there are limited empirical data regarding sleep disturbances among military-affiliated women.

Infant sleep problems further contribute to significant maternal sleep issues [30-32] and maternal mental health consequences, regardless of the mothers’ depression history [32-34]. Mothers who report poor infant sleep behavior have significantly more depressive symptoms than mothers who report good infant sleep [35-37]. There is some evidence that maternal sleep quality is a mediator of this relationship [35]. Persistent, rather than transient, infant sleep issues contribute to maternal depression, poor sleep, and poor family functioning well into toddlerhood [33,38,39]. We propose that focusing and intervening on infant sleep is a viable pathway to improve maternal sleep and well-being.

Here, we describe the protocol for a randomized controlled trial (RCT; Clinical Trials.gov CT94252410690; Sleep Health and Mood in Newly Expectant Military Mothers [SHINE]) with the following aims.

1. Determine whether the use of a smart bassinet (SB) augments infant sleep and improves maternal sleep during the first 6 months post partum compared to the use of a traditional bassinet (TB). (1) Hypothesis 1a: Infants who sleep in SB will have better sleep (ie, diary and Brief Infant Sleep Questionnaire as maternally reported) [40] than those who sleep in TB. Outcomes will include maternally reported sleep duration, awakenings, as well as objectively assessed (actigraphy) wake after sleep onset and sleep onset latency. (2) Hypothesis 1b: Mothers of infants who sleep in SB will have better subjectively reported (ie, diary and Pittsburgh Sleep Quality Index) and objectively assessed (actigraphy) sleep compared to those of infants who sleep in TB. Outcomes will be the same as those for infants.
2. Determine the effect of SB on maternal postpartum depressive and anxiety symptoms and evaluate the model that the association between SB and postpartum depressive symptoms is mediated by both infant and maternal sleep. (1) Hypothesis 2a: Mothers of infants who sleep in SB will have fewer postpartum depressive symptoms over time (ie, Edinburgh Postnatal Depression Scale) compared to those of infants who sleep in TB. (2) Hypothesis 2b: SB improves infant sleep, which then predicts better maternal sleep, and thereby predicts lower maternal depressive symptoms.
3. Compare the trajectory of immune system function from late pregnancy through post partum between participants with and without PMADs and between SB and TB groups. (1) Hypothesis 3a: Peripheral cytokines, immune cells, and stimulated cytokine profiles of women with PMADs assessed in late pregnancy and at 3 and 6 months post partum will indicate greater innate immune activity compared to those of women without PMADs. (2) Hypothesis 3b: Mothers of infants who sleep in SB will exhibit lesser innate immune dysfunction than mothers of infants who sleep in TB.
4. Exploratory aim: Evaluate whether the elevated risk demonstrated by previously identified PMAD epigenetic biomarkers at the TTC9B and HP1BP3 genes can be modified by using an SB. We hypothesize that the elevated risk will be reduced in the SB group compared to that in the TB group.

## Methods

### Study Design

**Figure 1:**
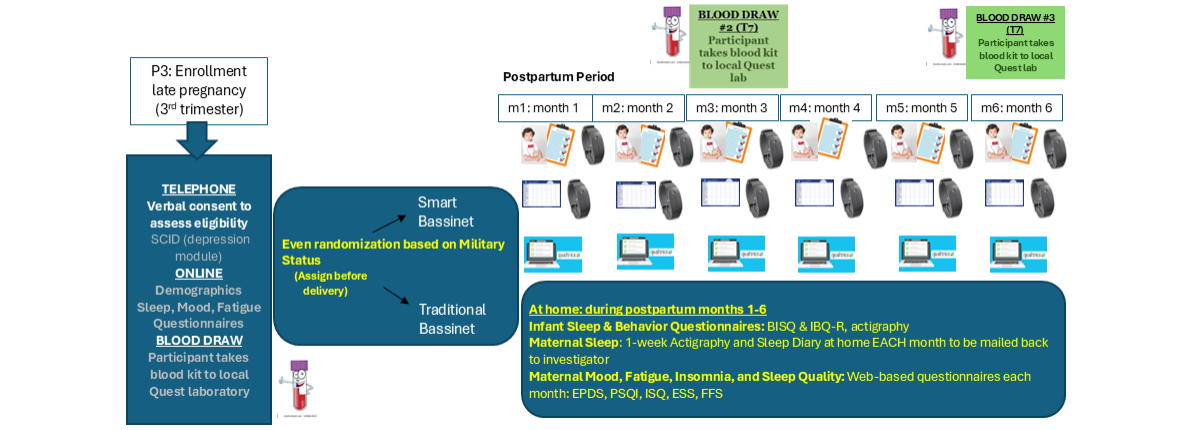
Flowchart in this study. BISQ: Brief Infant Sleep Questionnaire; EPDS: Edinburgh Postnatal Depression Scale; ESS: Epworth Sleepiness Scale; FFS: Flinders Fatigue Scale; IBQ-R: Infant Behavior Questionnaire-Revised; ISQ: Insomnia Symptom Questionnaire; PSQI: Pittsburgh Sleep Quality Index; SCID: Structured Clinical Interview for Diagnostic and Statistical Manual of Mental Disorders-5.

This study is a 2-arm, parallel-group RCT. Participants will be randomized at 1:1 ratio to either SB group or TB group. Outcomes are assessed at 7 timepoints (Figure 1): late pregnancy (P3) and in postpartum months 1-6 (M1-M6).

### Ethics Approval

This study has been approved by the institutional review board of the University of Colorado Colorado Springs (#2024-071) and the Office of Human Research Oversight (US Army Medical Research and Development Command). The SHINE trial was previously registered with Clinical Trials.gov (CT94252410690). This protocol follows recommendations from CONSORT (Consolidated Standards of Reporting Trials) 2010 statement checklist (Multimedia Appendix 1) and guide [41]. All participants will be required to provide informed written consent prior to enrollment. Participation is completely voluntary, and the participant has the right to withdraw at any time. All participants will be assigned a 6-digit ID number to be used so that all data collected will be deidentified. Participants will be compensated for their time. There are 7 timepoints in this study. At baseline (late pregnancy), participants will be compensated US $100; at postpartum months 1, 2, 4, and 5, they will be compensated US $50 for data collection, and at postpartum months 3 and 6, they will be compensated US $100. At baseline and at postpartum months 3 and 6, they will provide a blood sample.

### Participants

At the time of recruitment (P3) (third trimester), participants will complete a screening survey to determine their eligibility. Eligible pregnant women (N=342) will be recruited nationwide. The inclusion criteria were pregnant military-affiliated individuals with a singleton gestation; age 18-45 years; ability to communicate in English during the screening process; access to a computer, smartphone, or tablet with internet service; willing to use the bassinet they are randomized to; and willing to travel to a local Quest Diagnostics (an insert description) for blood draw. The exclusion criteria were presence of a depressive or anxiety disorder assessed over videoconference using the Structured Clinical Interview for Diagnostic and Statistical Manual of Mental Disorders-5-Clinical Version mood disorders section [42]; current active suicidal ideation, medical or psychiatric instability, or active substance abuse or dependence during the last 90 days; plans to co-sleep with infant; younger than 18 years or older than 45 years; multiple gestations; type 1 diabetes; congenital fetal anomalies; tobacco use (current); self-reported, untreated comorbid sleep disorders, including narcolepsy, periodic leg movement disorder, or obstructive sleep apnea; and current use of psychotropic or sleep medications.

### Procedures

#### Recruitment and Consent

Advertisement for this study will take place primarily online (social media) and through partner advocates (Postpartum Support International and Maternal Mental Health Leadership Alliance). Pregnant military-affiliated individuals who reside in the United States are eligible. Interested participants will contact the study coordinator for additional information and initial eligibility screening, including verification of military status.

#### Telephone Interview and Screening

Participants who meet initial inclusion criteria and have completed the consent and baseline questionnaires will be contacted for a telephone interview to assess further eligibility. The interview will contain items of the Structured Clinical Interview for Diagnostic and Statistical Manual of Mental Disorders-5-Clinical Version [42] and the Structured Clinical Interview for Sleep Disorders-Revised [43]. Eligible participants will be randomly assigned to either SB group or TB group. The SB will be shipped to the participants and returned after M6 at no expense to them. TB group participants will be shipped a standard bassinet (HALO BassiNest Swivel Sleeper) to keep. Mother-infant dyads (up to N=342) will be randomly assigned to conditions by using a random number generating program found on a widely used and reputable website [44]. We will perform 1:1 randomization. Variability in the length of bassinet usage may be impacted by size of infant, parental preference, or infant temperament. However, data will be collected through 6 months post partum.

#### Data Collection

For each assessment (Figure 1), all participants will receive an email with a REDCap (research electronic data capture) link for web-based data collection. All web-based survey data will be published in accordance with the CHERRIES (Checklist for Reporting Results of Internet E-Surveys) checklist. To mitigate and minimize the possibility of missing data in the measures, we will program electronic questionnaires to prevent skipping questions. REDCap is a web application and back-end database model designed to support data capture for research studies. REDCap is an open-source tool developed by Vanderbilt University to build and manage web-based forms for data collection. REDCap was developed specifically based on guidance from the Health Insurance Portability and Accountability Act of 1996 security guidelines and contains features such as data encryption. In addition, participants will receive a monthly package containing 2 actigraphs, a baby’s day diary, and a prepaid return envelope.

### Adverse Events

Adverse events will be monitored monthly via questionnaires and during telephone contact at month 3 and month 6 (phone calls to remind them to get blood drawn). Further, participants will be asked to report to the research team immediately if they experience unwanted adverse effects during participation. These events will be recorded and included in human research ethics reports. The Edinburgh Postpartum Depression Scale [45] has a single item that denotes suicidal ideation. It will be flagged such that if the participant endorses the item, a telephone call from a clinical psychologist (LF) to the participant will be initiated to conduct a risk assessment and assist with triage, as necessary.

### Measures and Materials

Table 1 describes the schedule of the study assessments for primary and secondary measures.

**Table 1:** Assessments and timeline.

Measures			Baseline enrollment		1 month PP^a^		2 months PP		3 months PP		4 months PP		5 months PP	6 months PP
**Maternal screening measures**														
	Consent/inclusion/exclusion	✓												
	Demographics	✓												
	SCID^b^ depression module/sleep SCID	✓												
	Infant assessments													
	Actigraphy			✓		✓		✓		✓		✓		✓
	Brief Infant Sleep Questionnaire			✓		✓		✓		✓		✓		✓
	Infant Behavior Questionnaire-Revised			✓		✓		✓		✓		✓		✓
	Baby Cry/Fuss chart			✓		✓		✓		✓		✓		✓
**Maternal assessments (primary)**														
	Sleep diary			✓		✓		✓		✓		✓		✓
	Actigraphy			✓		✓		✓		✓		✓		✓
	Pittsburgh Sleep Quality Index	✓		✓		✓		✓		✓		✓		✓
	Edinburgh Postnatal Depression Scale	✓		✓		✓		✓		✓		✓		✓
	Generalized Anxiety Scale	✓		✓		✓		✓		✓		✓		✓
	Tasso blood collection kit (Epigenetics)	✓												
	Blood samples (inflammation)	✓						✓						✓
**Maternal assessments (secondary)**														
	Insomnia Symptom Questionnaire	✓		✓		✓		✓		✓		✓		✓
	Epworth Sleepiness Scale	✓		✓		✓		✓		✓		✓		✓
	Flinders Fatigue Scale	✓		✓		✓		✓		✓		✓		✓
	Treatment expectations	✓						✓						✓
	Social support survey	✓		✓		✓		✓		✓		✓		✓
	Adherence questionnaire							✓						✓
	Delivery information			✓										

^a^PP: post partum.

^b^SCID: Structured Clinical Interview for Diagnostic and Statistical Manual of Mental Disorders-5.

#### Primary Infant Assessments

The mother will record infant sleep for 1 week per month in the baby’s day diary [46]. Boxes are filled in daily for 1 week with symbols that correspond to when the infant is asleep, awake, awake and fussy, sucking, or feeding. This measure has been widely used and exhibits desirable reliability and validity [47,48]. The Brief Infant Sleep Questionnaire [40] is used to assess the infant’s sleep during the past week. The Infant Behavior Questionnaire-Revised [49] will be used to allow mothers to report, on a 7-point scale, the frequency with which infants have enacted specific behaviors in common situations during the past week.

#### Primary Maternal Assessments

The Consensus Sleep Diary [50] will collect subjective sleep, while actigraphy will be contemporaneously used to derive objective sleep data. The Pittsburgh Sleep Quality Index [51] will measure habitual sleep quality. The Edinburgh Postnatal Depression Scale [52,53] will be used to assess changes in the depressive symptoms over time. The Generalized Anxiety Disorder Scale-7 items [54] and the Perinatal Anxiety Screening Scale [55] will be used to assess anxiety symptoms over time.

Participants will have their blood drawn (up to 20 mL) at enrollment, 3 months post partum, and 6 months post partum at a Quest Diagnostics laboratory near them (Figure 1). Briefly, blood collection kits with ethylenediaminetetraacetic acid tubes will be mailed to participants with a requisition form and a laboratory directive (from Quest) that provides the Quest Diagnostics Patient Service Center instructions on how to collect and process the study kit. Blood samples will be shipped in a temperature-controlled fashion to the Psychoneuroimmunology in Pregnancy & Postpartum (PIPPI) laboratory at Weill Cornell Medical College for analysis. Measurements of circulating cytokines, stimulated cell assays, and flow cytometry analysis will be conducted to evaluate the role of immune dysregulation.

#### Secondary Maternal Assessments

The Insomnia Symptom Questionnaire [56] is a self-report instrument that identifies a clinically relevant case definition of insomnia consistent with widely used insomnia classification criteria. It has 13 items, which identify sleep symptoms, frequency, duration, and related daytime impairment. Scoring indicates positive or negative for insomnia. The Epworth Sleepiness Scale [57] will assess daytime sleepiness. The Flinders Fatigue Scale [58] will assess daytime fatigue.

#### Measures in Both Mothers and Infants

Actigraphy watches (Phillips/Respironics and ActiGraph, LLC) will be provided to the dyad each month to collect objective sleep data. Data collection and interpretation will be consistent with previous reports [59-61]. For infants, watches will be placed on the right ankle in accordance with suggested guidelines [61-63].

### Epigenetic Biomarker Testing

#### Blood Draw Collection

Upon enrollment, participants will be sent supplies from TruDiagnostic for blood collection, including a Tasso blood collection kit and a prepaid mailing kit to TruDiagnostic. Blood samples will be processed using the Illumina Human MethylEPIC beadchip. Methylation data for the *TTC9B*, *HP1BP3*, and *MS4A7* genes will be sent to Dr Zach Kaminsky who will use the published algorithm for predicting postpartum depression risk to determine if the participant’s epigenetic methylation patterns are consistent with increased risk for postpartum depression. Participants will not be informed of their biomarker status, as we are still determining the clinical value of the biomarkers.

#### Immune Markers

Blood collection kits with ethylenediaminetetraacetic acid tubes will be mailed to participants with a requisition form and a laboratory directive (from Quest) that provides the Quest Diagnostics Patient Service Center instructions on how to collect and process the study kit. Aliquots of plasma and the second tube of whole blood will be shipped overnight to Weill Cornell Medicine. Peripheral blood mononuclear cell (PBMC) suspensions will be prepared within 24 hours of blood collection by low-density gradient centrifugation via Hermle Z300 (K#55085010) to avoid erythrophagy-related activation of the monocytes. PBMCs will be frozen and stored at –80 °C using 10% dimethylsulfoxide, thereby allowing all samples to be analyzed in parallel. Plasma will also be stored at –80 °C. In our prior studies, we have missing biological data (due to incomplete blood draws) in about 7% of the visits; we anticipate similar rates in this study.

#### Cytokine Analysis

Cytokine analysis will be performed under Dr Osborne’s supervision in the PIPPI laboratory at Weill Cornell Medicine. Plasma cytokines will be measured using the Meso-Scale Discovery Ultrasensitive Proinflammatory Multiplex kit (Meso Scale Diagnostics LLC) according to the manufacturer’s protocol in duplicate and read using the MS2400 imager (Meso Scale Diagnostics LLC). We will calculate the coefficient of variation for each woman’s replicates when both have concentrations above the limit of detection.

#### Cell Preparation and Stimulation

Maternal PBMCs will be thawed in Roswell Park Memorial Institute 1640 medium and distributed into three 24-well multiplates (5 × 10^5^ cells/well). Remaining cells will be aliquoted into two 5-mL culture tubes (2 × 10^6^ cells/tube). Cells will be maintained in a humidified atmosphere of 95% air/5% CO_2_ at 37 °C. After 24 hours, PBMCs in the 24-well plates will be treated with increasing concentrations of cortisol. Hydrocortisone stock solutions will be prepared by dissolving hydrocortisone in ethanol and then diluting with pyrogen-free sterile saline solution (NaCl 0.9%) to achieve equipotent final culture concentrations of 0.2 µg/mL (physiological plasma cortisol level) and 0.4 µg/mL (plasma cortisol level in high-stress status). PBMCs will be treated with a final cortisol concentration of 0, 0.2, and 0.4 µg /mL. Cells in the 5-mL culture tubes will be stimulated with phorbol myristate acetate (Sigma Aldrich) and ionomycin (Sigma Aldrich) in the presence of GolgiStop (Becton Dickinson) for 4 hours in 37 °C under a 5% CO_2_ environment to stimulate T-effector cells. Following the incubation periods, the supernatant from cortisol-stimulated cells will be collected and stored at –80 °C until assayed for interleukin (IL)-6, IL-10, IL-8, and IL-4 by using a commercial enzyme-linked immunosorbent assay. Cellular pellets will be resuspended and prepared for flow cytometry to assess the immune cell phenotype.

#### Flow Cytometry Analysis

Flow cytometry analysis will be performed on the BD LSRII instrument (BD Biosciences, 3 laser, 14 color) within the Cubillos-Ruiz laboratory adjacent to Dr Osborne’s PIPPI laboratory. Additional analyses, as necessary, will occur in the Weill Cornell Flow Cytometry Core, which houses a BD AriaII sorter, 5 laser (355, 405, 488, 561, 640) capable of 4-way sorting; a BD Influx sorter, 6 laser (355, 405, 445, 488, 561, 640) capable of 6-way sorting; a BD Fortessa analyzer, 5 laser (355, 405, 488, 561, 640) 20 parameter and a BD FACSCelesta analyzer, and 3 laser (405, 488, 561) 14 parameter. Panel one will include membrane staining markers and will be performed using thawed PBMCs. Stained cells will be analyzed by 8-color flow cytometry (with anti-CD3 BV786, anti-CD4 Pe-Cy7, anti-CD8 BV605, anti-CD14 antigen-presenting cell, anti-CD25 phycoerythrin, anti-CD16 PerCp-Cy5.5, anti-CD56 fluorescein isothiocyanate, and anti-CD19 BV421; Becton Dickinson). We will use a gating method to measure subsets of monocytes, lymphocytes, natural killer cells, T-cells, B-cells, CD4+ T helper cells, and CD8+ cytotoxic T cells. Samples stimulated with phorbol 12-myristate 13-acetate and ionomycin will be stained using a T-cell specific panel and analyzed by 8-color flow cytometry (with anti-CD3 BV786, anti-CD4 Pe-Cy7, anti-CD45RO BV605, anti-IFN-γ antigen-presenting cell, anti-CD25 PE, anti-FoxP3 PerCp-Cy5.5, anti-IL-4 fluorescein isothiocyanate, and anti-IL-17A BV421; Becton Dickinson).

### Process Measures

#### Treatment Expectation Questionnaire

An adapted version of the Treatment Expectation Questionnaire [64] will be administered at month 3 and month 6 to assess the mother’s expectations of benefits, satisfaction with condition, and fears. This measure asks about expectations regarding symptom relief, improvement, and benefits from using the specific bassinet. It allows for comparison of the impact of multidimensional expectations across different conditions. Open-ended comment boxes will be provided for mothers to report experiences, adverse events, or other comments.

#### Patient Adherence

Various items will be collected to determine patient adherence: (1) self-reported adherence assessed using an adapted survey checklist provided by Dr Stremler, (2) self-report data on bassinet usage, (3) pictures of bassinet use by participants, and (4) comment boxes to explain nonadherence.

#### Additional Factors to Consider and Use as Covariates

These include (1) presence of spouse/partner and other children at home [36,65-67]; (2) type of feeding [68-70]; (3) infant variables such as acid reflux, colic, and gestational age; (4) consistency of bedtime routine [71-73] and when moved to a crib; (5) history of childhood trauma in the mother via the Childhood Trauma Questionnaire [74], as prior trauma is highly correlated with depressive episodes, especially in new mothers [75,76] and negatively affects sleep [77]; and (6) type of delivery, complications, or time spent in neonatal intensive care unit.

### Statistical Analysis

#### Power

Power analyses were performed using *P*<.05 (2-tailed) at .80 power, based on recommended guidelines [78-80]. The power analyses were conducted for each statistical technique to be pursued in aims 1 to 3: mixed (between-subjects and within-subjects) multivariate analysis of variance (MANOVA), mixed (between-subjects and within-subjects) analysis of variance, independent 2-sided *t* test, dependent 2-sided *t* test, correlation, and the mediational models involving structural equation modeling (based on the β coefficient). The pilot study collected similar measures from pregnant/postpartum women with a history of major depressive disorder who used SB or HALO TB. Hence, the power analyses were based on pilot sleep and mood data. Calculations revealed an average effect size across all postpartum timepoints (T1 to T6) between the treatment and control groups on the following measures: infant sleep (*d*=0.39), postpartum sleep quality (*d*=0.27), and postpartum depression (*d*=0.33). Based on these effect sizes, a sample size of 208 participants is needed for infant sleep, 434 participants for postpartum sleep quality, and 286 participants for postpartum depression to attain statistical significance. Thus, a final sample size of 393 participants will be targeted (allowing for an attrition rate of 15%).

#### Randomization

AL generated the random sequence by using a random number generating program found on a widely used and reputable website [44]. We performed 1:1 randomization, stratifying for antidepressant use (actively taking or not). The study coordinator is responsible for the assignment based on the random sequence. This is not a blinded study, as there are distinct differences between the 2 bassinets.

#### Intention-To-Treat Analyses

Intention-to-treat analyses [81,82] will be conducted to understand the extent to which participants who satisfy the inclusion criteria for enrollment and are therefore randomized to one of the two conditions (intention-to-treat population) are similar to participants who provided complete data across the measurement rounds (actually treated sample). The purpose of intention-to-treat analyses is to scrutinize whether the conclusions drawn from the RCT remain robust even after considering participant attrition across time [81]. We will adhere to the recommended guidelines for performing multiple imputation on clinical research data [83].

#### Mean Differences

Initial analyses will be performed to verify that the random assignment procedure successfully equalized participants between the treatment and control groups on the preintervention variables. Thus, MANOVA will compare these 2 groups on the entire set of preintervention baseline measures. A chi-square test will verify that both groups have the same distribution of infant boys and girls. As participants will be randomized to groups using a random number generator, baseline equivalency of the 2 groups is anticipated.

Six separate mixed 2 (treatment vs control) × 6 (assessment period) MANOVA models will be conducted. Specifically, to assess hypothesis 1a (aim 1), the model (model 1) will be applied to the infant sleep quality outcome measures. To address hypothesis 1b (aim 1), the analysis (model 2) will be applied to maternal postpartum sleep quality outcome measures. To evaluate hypothesis 2a (aim 2), the model (model 3) will be applied to the maternal postpartum depression outcomes. To examine hypothesis 3a (aim 3), the model (model 4) will be estimated on the measures involving peripheral cytokines, immune cells, and stimulated cytokine profiles. To examine hypothesis 3b (aim 3), the model (model 5) will be undertaken on the measures of T-cell dysfunction. Furthermore, to examine the exploratory aim, the model (model 6) will be estimated on the epigenetic biomarkers.

In each of the mixed MANOVA models, several follow-up analyses will be conducted to examine the results. First, a mixed (between-subjects and within-subjects) analysis of variance will be applied on each specific outcome. Second, independent 2-sided *t* tests will assess mean differences between the treatment and control group (at each timepoint) on each outcome. Third, dependent 2-sided *t* tests will permit scrutiny of the temporal change in mean scores between every 2 timepoints on every outcome. Fourth, the longitudinal trajectories across the entire 6-month period for both the treatment and control groups will be graphed using 95% CIs to provide a visual tool to offer insights to understand the effectiveness of the RCT.

Prior to conducting analyses, the normal distribution of all the measures will be inspected. If a variable is evidenced to be nonnormally distributed, then the bootstrapping variant of these statistical techniques instead will be performed to obtain corrected *P* values [84].

### Mediational Analyses

**Figure 2:**
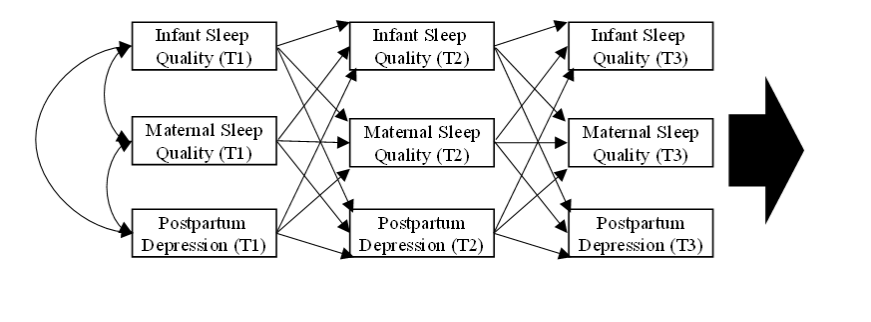
Cross-lagged panel model. For diagrammatic clarity, not displayed but will be estimated as part of the model are the constructs for postpartum months 3 (T4), 4 (T5), and 5 (T6), the correlations across constructs in every measurement round, and predictive error terms.

Cross-lagged panel designs will be pursued to make inferences about the temporal priority of a set of measures assessed longitudinally [85-87]. Accordingly, cross-lagged panel models permit the opportunity to determine the temporal precedence of antecedents and consequents in the set of constructs [86]: infant sleep quality, maternal sleep quality, and postpartum depression. The depiction of the proposed cross-lagged panel model to be tested is shown in Figure 2.

**Figure 3:**
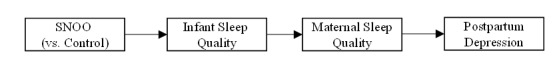
Mediational model. For diagrammatic clarity, not displayed but will be estimated as part of the model are the predictive error terms. SNOO: smart bassinet.

The cross-lagged panel model findings in the above model are expected to help inform how to specify the mediational model for hypothesis 2b. We propose that the cross-lagged panel model will evidence the following significant temporal cross-lagged directions that are consistent with hypothesis 2b: SB versus TB→infant sleep→maternal sleep→postpartum depression. Thus, a longitudinal mediational model, incorporating the intervention factor, will be specified and estimated (Figure 3). This mediational model tests and proposes that following underlying mechanism: SB group (vs the control group) will improve infant sleep quality, which would then predict better maternal sleep quality and finally contribute to lower maternal postpartum depression. All the outcomes of aim 1 are incorporated into the aim 2 mediational model to provide comprehensive and compelling insights that will statistically connect all the psychosocial constructs and understand the underlying process of this RCT.

The cross-lagged panel model and the meditational model each will be estimated with structural equation modeling [88]. The overall adequacy of these structural equation models will be assessed using fit indices [89-91]. A nonsignificant model chi-square test is desired to signify that the model approximates the underlying data, but this index tends to be sensitive to sample size [92]. Thus, additionally interpreted will be the comparative fit index and the Tucker-Lewis index, with higher values, preferably above 0.90, indicative of a good-fitting model [93,94]. The root mean-square error of approximation is sufficiently sensitive in detecting model misspecifications and provides a 90% CI [95]. Values below 0.05 indicate close fit, between 0.05 and 0.08 fair fit, between 0.08 and 0.10 mediocre fit, and above 0.10 poor fit [95].

If the fit indices for a model would be judged satisfactory, then the magnitude of the direct paths and the *P* values of these paths will be interpreted [96]. In the mediational model, the statistical significance of these mediational processes starting from the SB intervention (vs control) to infant sleep quality to maternal sleep quality and finally to postpartum depression with be evaluated in tests of indirect effects to determine whether the mediational process is significant beyond chance [88]. As recommended for structural equation models [96], bootstrapping with bias-corrected CIs will be applied to adjust for potential nonnormality of the variables.

### Statistical Analyses for Immune Measurements

The initial statistical approach will include a descriptive cross-sectional analysis at each timepoint, both for quality assurance and to attain a better understanding of the distributions of the immune cell and cytokine profile variables for use in subsequent analyses, including the need for any transformations (ie, logarithmic transformation to symmetrize the residuals). Factor analysis will be employed as a means of data reduction to probe latent cytokine profiles reflected across the manifest indicators (eg, tumor necrosis factor-α, IL-6, IL-1β). We will first compare levels of a combined score of peripheral innate immune cytokines and frequency of maternal immune cells between women who do and do not develop PMADs in separate models to evaluate cross-sectional effects, employing a multivariable linear regression model to adjust for relevant covariates (eg, gestational age, BMI). Similar models will be repeated for maternal cytokines and innate cell recruiting factors as produced by the stimulated cells. Second, we will test whether the magnitude of group difference varies across the study period in a longitudinal analysis. We will fit a generalized linear population-average model estimated using generalized estimating equations with exchangeable working correlation structure to account for within-women correlation of mood measures. The model will include the relevant immune markers identified in the cross-sectional analyses, indicators for visit and group, as well as any relevant interaction terms (immune marker by group or immune marker by visit). The models will adjust for baseline severity of depressive symptoms and psychotropic medication use. Standard model checking will be performed, and the most parsimonious model will be selected based on the Akaike information criterion or quasi-likelihood information criterion for generalized estimating equations models. To test the within-group effects of severity of PMAD symptoms, the dichotomous group variable will be replaced by continuous Edinburgh Postpartum Depression Scale and Generalized Anxiety Disorder Scale-7 item scores in analytic models and restricted to only the subset of women with significant PMAD symptoms.

### Missing Data

Attrition in our prior studies has been approximately 15%, and we have accounted for that in our planned sample size. In addition, in our prior studies, we have missing biological data (due to incomplete blood draws) in about 7% of the visits; we anticipate similar rates in this study, which will use the same approach. Given the prospective nature of our study, we will carefully evaluate missingness. If identified, we will employ one of the two strategies to handle missing data. The first approach relies on a technical assumption of missing at random and involves inclusion of covariates related to missingness in all analyses. Full information maximum likelihood performs well under ignorable missing data conditions such as missing at random. A second strategy we will use is multiple imputation. Although this has the potential to yield biased estimates dependent on the nature of missingness [97], we will employ this approach to implement sensitivity analysis under plausible assumption of missing not at random to examine the robustness of our inference [98].

## Results

This project was funded in June 2024 and approved by the institutional review board of University of Colorado Colorado Springs (#2024-071) and by the Office of Human Research Oversight in February 2024. Recruitment for this study began in January 2025. We anticipate that the data analysis for the primary and secondary aims will be completed by July 2028. The methodology for the web-based surveys will be reported according to the CHERRIES checklist before the submission of any manuscript. The results from this trial will be used to extend SB efficacy in larger military cohorts and in women experiencing active perinatal mood disorders.

## Discussion

### Implications of SHINE

At the conclusion of this project, we will have gained a thorough understanding of how SB affects infant and maternal sleep compared to TB and its association with maternal mood through 6 months post partum in military-affiliated women. We will also significantly extend our current knowledge regarding the biological mechanisms associated with PMADs and how they may be modified by behavioral interventions. Future directions will include evaluations of women with current depressive disorders, women with a history of depression but not actively depressed, women with and without a history of trauma, and women who deliver prematurely to determine if SB can mitigate depressive symptomatology over the course of 12 months. This is a primary goal, as PMAD is a serious public health concern.

### Limitations

This study will, however, have important limitations that warrant discussion. The study goal is to determine whether SB can prevent or reduce the reoccurrence of PMADs and not for use as an active treatment. Hence, we chose to exclude participants with active psychopathology at enrollment. We focused on at-risk participants. Those with active psychopathology are not at-risk—they have it. Moreover, we felt that it was a matter of safety. Recruiting participants from across the United States restricts the ability of our team to manage or treat an actively depressed mother. Ethically, we believe that we needed to demonstrate that SB can mitigate or prevent PMADs before evaluating whether it can treat active PMADs. However, if a participant develops PMAD at post partum, they will remain in the study and be referred for treatment. To clarify, we are only excluding women with a positive diagnosis of SCID; women with a history of PMAD or who are effectively treated will be included. This decision comes with some concerns. One is that we may have too small of a magnitude or range of mood symptoms to observe a true positive effect of SB on mood symptoms. In other words, the population will be too mentally normal/healthy. We believe, however, that the diverse sample will provide enough variability in mood symptoms, especially since a greater percentage of active-duty women and military spouses experience mental health conditions and at a higher rate than civilians [99-101]. If we notice that the rate of PMAD symptoms is limited or if we are excluding too many participants due to active psychopathology, we may alter the design to include active psychopathology. Another limitation is that the study requires participants to have access to the internet; therefore, individuals of low socioeconomic status and certain racial/ethnic groups may potentially be excluded from the study [102]. However, low-income Americans have made gains in technology and access to the internet, which is likely to continue in the future. A primary goal of this study is to have a diverse sample of racial and ethnic backgrounds. Recruiting and retaining a broad range of participants, particularly those of low socioeconomic status, has been challenging, with recent calls to enhance representation [103,104]. In order to provide an equitable interpretation of the data, the participants need to be diverse and accurately reflect the population of the United States. Therefore, a major emphasis will be on recruiting a diverse and representative sample of military-affiliated participants. Although attrition has been a minimal issue in our studies, we are cognizant of the duration and expectations of the protocol. Thus, our group will maintain regular contact and troubleshoot, if necessary, to enhance retention.

### Conclusions

Pregnant active-duty and military spouses are an underserved population. Data suggest that persistent, rather than transient, infant sleep issues contribute to maternal depression, poor maternal sleep, and poor family functioning. We contend that to achieve the goal of reducing PMADs among new military-affiliated mothers, sufficient and efficient sleep is crucial. Understanding that there are few nonpharmacologic interventions that significantly improve infant or maternal sleep highlights the need for these data. This will be the first study to assess sleep methodically and longitudinally from infants who sleep in an SB and their mothers versus those who sleep in a TB and their mothers and how sleep from both individuals interacts to affect postpartum mood. We hypothesize that this study will indicate that SB may indeed reduce PMADs in military-affiliated mothers.

## Appendix

Multimedia Appendix 1 CONSORT (Consolidated Standards of Reporting Trials) 2010 statement checklist.

Multimedia Appendix 2 Peer review from the Congressionally Directed Medical Research Programs - Fiscal Year 2023 Peer Reviewed Medical Research Program - Lifestyle and Behavioral Health Interventions Research Award - Clinical Trial (Department of Defense, U.S. Army Medical Research and Development Command, USA).

## Abbreviations

CHERRIES: Checklist for Reporting Results of Internet E-Surveys
CONSORT: Consolidated Standards of Reporting Trials
IL: interleukin
MANOVA: multivariate analysis of variance
PBMC: peripheral blood mononuclear cell
PIPPI: Psychoneuroimmunology in Pregnancy & Postpartum
PMAD: postpartum mood and anxiety disorder
RCT: randomized controlled trial
REDCap: research electronic data capture
SB: smart bassinet
SHINE: Sleep Health and Mood in Newly Expectant Military Mothers
TB: traditional bassinet

## Data Availability

Data supporting this study will be openly available from the National Sleep Research Resource, which is a National Heart Lung Blood Institute–supported repository for sharing sleep data (polysomnography, actigraphy, and questionnaire-based) collected on tens of thousands of individuals from cohort studies, clinical trials, and other data sources.
