# Supplementary material for: Effects of Using a Smart Bassinet on the Mental Health of Military-Affiliated Pregnant Women: Protocol for a Randomized Controlled Sleep Health and Mood in Newly Expectant Military Mothers (SHINE) Trial
Source: JMIR Res Protoc. 2025 Apr 10;14:e66439. doi: 10.2196/66439 (PMC12022534; doi:10.2196/66439)
Supplement: Multimedia Appendix 2 [file resprot_v14i1e66439_app2.pdf]

AWARD/CONTRACT

1. THIS CONTRACT IS A RATED ORDER UNDER DPAS (15 CFR 700)	RATING	PAGE OF PAGES 1	7

2. CONTRACT (*Proc. Inst. Ident.*) NO. HT94252410690	3. EFFECTIVE DATE 15 Jun 2024	4. REQUISITION/PURCHASE REQUEST/PROJECT NO. 0012071533-0001

5. ISSUED BY CODE HT9425<br>ARMY MED RES ACQ ACTIVITY<br>808 SCHREIDER ST<br>FORT DETRICK MD 21702	6. ADMINISTERED BY (*If other than Item 5*) CODE<br>**See Item 5**

7. NAME AND ADDRESS OF CONTRACTOR (*No., street, city, county, state and zip code*)<br>THE REGENTS OF THE UNIVERSITY OF COLORAD<br>1420 AUSTIN BLUFFS PKWY<br>COLORADO SPRINGS CO 80918-3733	8. DELIVERY<br>[ ] FOB ORIGIN [X] OTHER (*See below*)<br>9. DISCOUNT FOR PROMPT PAYMENT<br>Net 30 Days<br>10. SUBMIT INVOICES 1 (*4 copies unless otherwise specified*) TO THE ADDRESS SHOWN IN: ITEM
CODE 2T882 FACILITY CODE	

11. SHIP TO/MARK FOR CODE HT0989<br>CONG DIR MED RES PRGM / CDMRP - MM<br>1077 PATCHEL STREET<br>FORT DETRICK MD 21702	12. PAYMENT WILL BE MADE BY CODE HQ0490<br>DEFENSE FINANCE AND ACCOUNTING SERVICE<br>DFAS-INDY VP GFEBS<br>8899 E 56TH STREET<br>INDIANAPOLIS IN 46249-3800
13. AUTHORITY FOR USING OTHER THAN FULL AND OPEN COMPETITION:<br>[ ] 10 U.S.C. 2304(c)( ) [ ] 41 U.S.C. 253(c)( )	14. ACCOUNTING AND APPROPRIATION DATA<br>**See Schedule**

15A. ITEM NO.	15B. SUPPLIES/ SERVICES	15C. QUANTITY	15D. UNIT	15E. UNIT PRICE	15F. AMOUNT
	**SEE SCHEDULE**				
				**15G. TOTAL AMOUNT OF CONTRACT**	**$3,800,334.00**

16. TABLE OF CONTENTS

(X)	SEC.	DESCRIPTION	PAGE(S)	(X)	SEC.	DESCRIPTION	PAGE(S)
		**PART I - THE SCHEDULE**				**PART II - CONTRACT CLAUSES**	
X	A	SOLICITATION/ CONTRACT FORM	1		I	CONTRACT CLAUSES	
	B	SUPPLIES OR SERVICES AND PRICES/ COSTS				**PART III – LIST OF DOCUMENTS, EXHIBITS AND OTHER ATTACH.**	
	C	DESCRIPTION/ SPECS./ WORK STATEMENT			J	LIST OF ATTACHMENTS	
	D	PACKAGING AND MARKING				**PART IV - REPRESENTATIONS AND INSTRUCTIONS**	
	E	INSPECTION AND ACCEPTANCE			K	REPRESENTATIONS, CERTIFICATIONS AND OTHER STATEMENTS OF OFFERORS	
	F	DELIVERIES OR PERFORMANCE					
	G	CONTRACT ADMINISTRATION DATA			L	INSTRS., CONDS., AND NOTICES TO OFFERORS	
	H	SPECIAL CONTRACT REQUIREMENTS			M	EVALUATION FACTORS FOR AWARD	

CONTRACTING OFFICER WILL COMPLETE ITEM 17 (SEALED-BID OR NEGOTIATED PROCUREMENT) OR 18 (SEALED-BID PROCUREMENT) AS APPLICABLE

17. [ ] CONTRACTOR'S NEGOTIATED AGREEMENT Contractor is required to sign this document and return copies to issuing office.) Contractor agrees to furnish and deliver all items or perform all the services set forth or otherwise identified above and on any continuation sheets for the consideration stated herein. The rights and obligations of the parties to this contract shall be subject to and governed by the following documents: (a) this award/contract, (b) the solicitation, if any, and (c) such provisions, representations, certifications, and specifications, as are attached or incorporated by reference herein. (Attachments are listed herein.)	18. [X] SEALED-BID AWARD (Contractor is not required to sign this document.) Your bid on Solicitation Number REF: See Section 00800 including the additions or changes made by you which additions or changes are set forth in full above, is hereby accepted as to the terms listed above and on any continuation sheets. This award consummates the contract which consists of the following documents: (a) the Government's solicitation and your bid, and (b) this award/contract. No further contractual document is necessary. (Block 18 should be checked only when awarding a sealed-bid contract.)
19A. NAME AND TITLE OF SIGNER (*Type or print*)	20A. NAME OF CONTRACTING OFFICER<br>JOSHUA MCKEAN / GRANTS OFFICER<br>TEL: 301-619-4046 EMAIL: joshua.d.mckean3.civ@health.mil

19B. NAME OF CONTRACTOR<br>BY<br>(*Signature of person authorized to sign*)	19C. DATE SIGNED	20B. UNITED STATES OF AMERICA<br>BY<br>(*Signature of Contracting Officer*)	20C. DATE SIGNED<br>06-Jun-2024

Section 00010 - Solicitation Contract Form

ITEM NO	SUPPLIES/SERVICES	QUANTITY	UNIT	UNIT PRICE	AMOUNT
0001					$3,800,334.00
	Proposal Log# PR230578<br>COST<br>Award Mechanism: FY23 Peer Reviewed Medical Research Program (PRMRP) Lifestyle and Behavioral Health Interventions Research Award - Clinical Trial<br>Sponsoring Agency: The Assistant Secretary of Defense for Health Affairs endorsed by the Department of Defense<br>FOB: Destination<br>PURCHASE REQUEST NUMBER: 0012071533-0001<br>PSC CD: AN11				
				ESTIMATED COST	$3,800,334.00
	ACRN AA<br>CIN: GFEBS001207153300001				$3,800,334.00

DELIVERY INFORMATION

CLIN	DELIVERY DATE	QUANTITY	SHIP TO ADDRESS	DODAAC / CAGE
0001	POP 15-JUN-2024 TO 14-JUN-2028	N/A	CONG DIR MED RES PRGM / CDMRP - MM<br>1077 PATCHEL STREET<br>FORT DETRICK MD 21702<br>FOB: Destination	HT0989

Section 00800 - Special Contract Requirements

ACCOUNTING AND APPROPRIATION DATA

AA: 09720232024013000018170440400410 R.0004371.23.1.26 6100.9000021001
COST CODE: AHPWB
AMOUNT: $3,800,334.00

ACRN	CLIN/SLIN	CIN	AMOUNT
AA	0001	GFEBS001207153300001	$3,800,334.00

CLAUSES INCORPORATED BY FULL TEXT

U.S. ARMY MEDICAL RESEARCH ACQUISITION ACTIVITY
AWARD SPECIFIC RESEARCH TERMS AND CONDITIONS

DIVISION I – AWARD COVER PAGES

A. Award Information

1. **Department of Defense Awarding Office:** USAMRAA
2. **Award number:** HT9425-24-1-0690
3. **Type of Award:** Grant
4. **Type of Award Action:** New
5. **i. Brief description of project or program:** Improving Maternal Mental Health in Military-Affiliated Pregnant Women: Effectiveness of a Smart Bassinet
   ii. Funding Overview

	Federal funds	Cost Sharing	Total amount
a. Obligated or deobligated this action	$3,800,334	N/A	$3,800,334
b. Cumulative obligations to date, including this and previous actions	$3,800,334	N/A	$3,800,334
c. Planned project costs in the currently approved budget through the end of the period of performance, to include any future incremental funding obligations	$3,800,334	N/A	$3,800,334
d. Total value, which includes any unexercised options for which amounts were established in the award	$3,800,334	N/A	$3,800,334

6. **Obligation/Effective Date:** See SF-26, Block 20c.
7. **Period of performance:** 6/15/2024 - 6/14/2028
8. **Authorities:** This award is made under the authority of 10 U.S.C. 4001.

9. **Assistance Listing Number:** 12.420-Military Medical Research and Development
10. **Project Performance Information:**
    i. This award is for research and development.
    ii. Scope of Work and Approved Budget:
        a. Specific Aims (Insert Specific Aims of the Project)
            - Specific Aim 1: Determine the effect of the SB on infant sleep and maternal sleep.
            - Specific Aim 2: Determine the effect of the SB on maternal postpartum depressive symptoms and evaluate the model that the association between the SB and postpartum depressive symptoms is mediated by both infant and maternal sleep.
            - Specific Aim 3: Compare trajectory of immune system function from late pregnancy through postpartum between PPD and non-PPD and between SB and TAU groups.

        b. Approved Budget:

	Year 1	Year 2	Year 3	Year 4
Total Costs	$980,848	$957,327	$983,810	$878,349

    iii. The following terms and conditions are incorporated herein by reference:
        a. Division III - USAMRAA Addendum to the DoD R&D General Terms and Conditions available at https://usamraa.health.mil/Pages/Resources.aspx
        b. The DoD R&D General Terms and Conditions (latest version), available at: https://www.nre.navy.mil/work-with-us/manage-your-award/manage-grant-award/grants-terms-conditions
    iv. These USAMRAA Award Specific Research Terms and Conditions are in addition to the terms and conditions incorporated above. Any inconsistencies in the requirements of this award will be resolved in the following order:
        a. Federal statutes
        b. Federal regulations
        c. 2 CFR part 200 with amendments, supplemented by DoD's implementation found in 2 CFR part 1104
        d. Division II - USAMRAA Award Specific Research Terms and Conditions
        e. Division III – USAMRAA Addendum to the DoD R&D General Terms and Conditions
        f. DoD R&D General Terms and Conditions

    v. **Grants Administration Office**

Grants Management Specialist (GMS):
Phone: 301-619-4019
Email: Darrell.L.Beaver4.civ@health.mil
Grants Branch Email: usarmy.detrick.medcom-usamraa.mbx.aa1@health.mil

    vi. **Grants Officer's Representative (GOR)**

Congressionally Directed Medical Research Program Office
Phone: 301-619-7071
Email: adam.a.book.civ@health.mil

B. Recipient Information

1. **Unique Entity Identifier:** RH87YDXC1AY5
2. **Recipient Business Name and Address:** The Regents of the University Of Colorado, 1420 Austin Bluffs Parkway, Colorado Springs, Colorado 80918-3733

3. **Name and Title of Authorized Representative:** Gwendolyn Gennaro
    a. Phone: 719-255-3153
    b. Email: osp@uccs.edu
4. **Principal Investigator (PI) and Organization:** Michele Okun
    a. Phone: 412-302-8030
    b. Email: mokun@uccs.edu
5. **Recipient's Indirect Cost Rate at the Start of the Performance Period:**
    Rate, Type, Basis, Period: 46%, Predetermined, MTDC, 7/1/2020-6/30/2024
    Negotiating Agency: DHHS

C. Additional Information:

1. **Award Modification:** The only method by which the award may be modified is by a formal, written modification signed by the USAMRAA Grants Officer. No other communications, whether oral or in writing, are valid to change the terms and conditions of this award. Awards will not be modified to provide additional funds for such purposes as reimbursement for unrecovered indirect costs resulting from the establishment of final negotiated rates or for increases in salaries, fringe benefits, changes in exchange rates, or other costs.
2. **Expiration of Funds**: Funds obligated on this award are available for use for a limited period based on the fiscal year (FY) of the funds. That time is considered when establishing your period of performance. **This award is funded with FY23 funds in the amount of $3,800,334 (CLIN 0001) which will expire for use on September 30, 2029.** You must monitor the established milestones, timelines, expenditures, and invoicing to make sure the project is on schedule and that you voucher promptly. **Final vouchers for expiring funds must be submitted at least 30 days prior to September 30, 2029**. If you have not submitted a final grant voucher and been paid before the expiration date of these funds, any excess funds will be deobligated from the award at that time.

DIVISION II – AWARD SPECIFIC RESEARCH TERMS AND CONDITIONS

Clinical Trial Registry

Certain clinical trials are required by U.S. law to be registered on the National Institutes of Health database entitled "ClinicalTrials.gov." For those trials required to be registered (see http://prsinfo.clinicaltrials.gov/, "Support Materials, including Data Element Definitions"), PIs must register clinical trials individually on http://www.clinicaltrials.gov. PIs must use a Secondary Protocol ID number designation of "CDMRP-Proposal Log Number" (e.g., CDMRP-PR230578). If several protocols exist under the same application, the Secondary Protocol ID number must be designated "CDMRP-Proposal Log Number-A, B, C, etc." (e.g., CDMRP-PR230578A). Clinical trials must be registered prior to enrollment of the first patient. Failure to do so may result in a civil monetary penalty and/or the withholding or recovery of award funds as per U.S. Public Law 110-85. For applicable Phase III clinical trials, PIs shall submit results of analyses of group differences on the basis of sex/gender, race, and/or ethnicity to clinicaltrials.gov at the time of final report submission. If final analyses of sex/gender and race/ethnicity are not available at the time of the final technical report, a justification and plan ensuring completion and reporting of the analyses should be submitted to USAMRAA.

Electronic Payment Instructions

i. The Procurement Integrated Enterprise Environment (PIEE) e-Business Suite is the required method to electronically process your requests for payments. Once on the PIEE e-Business Suite web site, select the Wide Area Workflow (WAWF) button to electronically submit "grant vouchers" (used for both grants and cooperative agreements). You must (i) register to use WAWF at https://wawf.eb.mil and (ii) ensure an electronic business point of contact (POC) is designated in the System for Award Management (SAM) site at https://www.sam.gov within ten (10) calendar days prior to requesting a payment for this award. The Award specific Research Terms and Conditions will include additional instructions on how to submit grant vouchers and who to contact for assistance if needed.

ii. Questions concerning specific payments should be directed to the Defense Finance and Accounting Service (DFAS), Indianapolis, at 1-888-332-7366, unless a different office is specified in Division II in your award specific terms and conditions. **You can also access payment and receipt information using the "myInvoice" button in PIEE at** https://wawf.eb.mil. The award number or grant voucher number will be required to inquire about the status of the payment.

iii. The following codes and information are required to initiate the grant voucher and assure successful flow of PIEE documents.

TYPE OF DOCUMENT: **Grant Voucher** ***(Used for both grants and cooperative agreements)***

CAGE CODE: **Enter Your Cage Code**

ISSUE BY DODAAC: **HT9425**

ADMIN BY DODAAC: **HT9425**

INSPECT BY DODAAC: **HT9425**

GRANT APPROVER DODAAC: **HT0983**

SHIP TO DODAAC: **HT9425**

LOCAL PROCESSING OFFICE DODAAC: **Not Applicable**

PAYMENT OFFICE FISCAL STATION CODE: **Unless otherwise specified in Division II in your award specific terms and conditions enter Fiscal Station DODAAC as HQ0490 = DFAS Indianapolis**

EMAIL POINTS OF CONTACT LISTING:
INSPECTOR: **Submit to Grants Branch Email identified in the Division I, 10.v.**
ACCEPTOR**: Submit to Grants Branch Email identified in the Division I, 10.v.**
RECEIVING OFFICE POC: **Submit to Grants Branch Email identified in the Division I, 10.v.**
GRANT ADMINISTRATOR: **Leave Blank**
GRANTS OFFICER: **Leave Blank**
ADDITIONAL CONTACT: **Submit to Grants Branch Email identified in the Division I, 10.v.**

Quarterly Technical Reports

a. For each year of the award, you must submit Quarterly Technical Progress Reports covering research results (positive and negative data) over a three-month period (quarter). A reporting quarter begins with the start date of the award and restarts annually from that date for the entire period of performance. A Quarterly Technical Progress Report for the fourth quarter each year is not required, as the Annual Technical Report must incorporate all four quarters of progress.

b. Quarterly reports are the most immediate and direct contact between you and the GOR. The reports provide the means for keeping the US Army Medical Research and Development Command (USAMRDC) advised of developments and problems as the research effort proceeds. The reports also provide a measure against which funding decisions are made.

c. Prepare all Quarterly reports in accordance with the Quarterly Technical Progress Report format, available at https://usamraa.health.mil/Pages/Resources.aspx. Each item of the report format must be completed.

d. Each report must be submitted electronically, within 30 days after the end of each quarter, through the Electronic Biomedical Research Application Portal (eBRAP) at https://ebrap.org/eBRAP/public/index.htm.

Name your file with your award number, followed by Year X Quarter Y Report (example: HT94252410690 Year 1 Quarter 1 Report.) If you have questions, contact the GOR.

Special Reporting Requirements for Annual/Final Technical Reports

Special Reporting Requirements for Annual/Final Reports (must be submitted as an appendix to the annual/final report)

Award Expiration Transition Plan: The Award Expiration Transition Plan (available on https://ebrap.org/eBRAP/public/Program.htm) must be submitted as an appendix to the final report.

Inclusion Enrollment Report: Inclusion enrollment data distributed on the basis of sex/gender, race, and ethnicity must be updated and submitted as an appendix to the annual and final report. The Public Health Service Inclusion Enrollment Report is available on the "Funding Opportunities & Forms" web page (https://ebrap.org/eBRAP/public/Program.htm) in eBRAP.

Subaward Costs

This award contains funds for one or more subawards. As the direct and primary recipient of USAMRAA grant funds, you are accountable to USAMRAA for the project performance, the appropriate expenditures of grant funds by all parties, and all other obligations of the recipient, as specified in Part 7, Subawards, of the DoD R&D General Terms and Conditions. In general, the requirements that apply to the recipient, also apply to the subrecipient (s).
